# Fabrication and Characterization of Physical and Mechanical Properties of Carbon Nanotubes—Graphene-Based Sandwich Composite Pressure Sensor

**DOI:** 10.3390/nano11051284

**Published:** 2021-05-13

**Authors:** Asar Ali, Farman Ali, Ahmad Rashedi, Ammar Armghan, M. R. Nurul Fajita, Fayadh Alenezi, N. B. Karthik Babu

**Affiliations:** 1 Department of Electrical Engineering, Qurtuba University of Science & Information Technology, Dera Ismail Khan 2950, Pakistan; engrasar45@qurtuba.edu.pk (A.A.); drfarmanali.optics@qurtuba.edu.pk (F.A.); 2 College of Engineering, IT and Environment, Charles Darwin University, Casuarina, NT 0810, Australia; 3 Department of Electrical Engineering, College of Engineering, Jouf University, Sakaka 42421, Saudi Arabia; aarmghan@ju.edu.sa (A.A.); fshenezi@ju.edu.sa (F.A.); 4 Bioresource Technology, School of Industrial Technology, Universiti Sains Malaysia, Penang 11800, Malaysia; fazita@usm.my; 5 Department of Mechanical Engineering, Centurion University of Technology and Management, Odisha 761211, India; karthikbabunitt@gmail.com

**Keywords:** carbon nanotubes, graphene, piezoresistive pressure sensors, resistance, composites

## Abstract

In this work, piezoresistive properties of graphene-multiwalled carbon nanotubes (MWCNTs) composites are investigated, characterized, and compared. Sandwich-type composite piezoresistive pressure-sensitive sensors (Ag/Graphene-MWCNT/Ag) with the same diameters, but different fabrication pressures and thicknesses were fabricated using the mortar and pestle/hydraulic press technique. To produce low-electrical-resistance contacts, both sides of the composite sensors were painted with silver (Ag) paste. All the sensors showed reductions in the direct current (DC) resistance ‘R’ with an increment in external uniaxial applied pressure. However, it was observed that higher fabrication pressure led to a lower resistance value of the composite, while the thicker samples give lower electrical conductivity and higher resistance than the thinner samples. The experimental data for all composite pressure sensors were in excellent agreement with the simulated results.

## 1. Introduction

Due to their characteristic specific modulus and specific strength properties, composite materials are nowadays widely used in many industries [1,2]. Nanocomposites are a group of materials within composite families that differ from conventional composites as a result of exceptionally high surface-to-volume ratios of the reinforcing phase and/or its exceptionally high aspect ratio. These nanocomposites recently attracted the focus of scientists and researchers due to their extraordinary physical, optical, electrical, mechanical, and piezoresistive properties. Graphene and multi-walled carbon nanotubes (MWCNTs) are the two most significant representatives of nanostructured materials [3]. The density of MWCNT is about 25–30% of steel, while its tensile strength and elasticity are 100 and 5 times greater than steel, respectively [4,5]. On the other hand, the Young’s Modulus of graphene is 1000 and 600 times larger than metals and semiconductors, respectively [6,7]. Graphene and MWCNTs are optimal nanostructured materials for pressure-sensing technology, due to their best structural properties, low density, high gauge factor, and a wide range of applications in many fields [8,9]. Therefore, graphene and MWCNT-based nanostructured materials have been utilized in various electronic devices like humidity sensors, strain sensors, displacement sensors, solar cells, temperature sensors, piezoresistive pressure sensors, etc. [10]. Pressure sensors can be fabricated on the basis of piezoresistive, capacitive, and inductive phenomena [11,12]. However, the piezoresistive pressure sensors outperform the others in daily life applications due to their simple device structure, easy signal collection, and low-cost and easy fabrication process [13]. Piezoresistive pressure sensors could be used in the aviation and automotive industries, touchscreen devices, and in almost every field of monitoring and sensing technology [14]. Therefore, scientists and researchers have extensively focused on the piezoresistive properties of the pressure sensors in recent years. For instance, Xing Chen investigated graphene piezoresistors as strain-gauge sensors and experimentally measured a very high gauge factor of around 150 [15]. In 2017, P. Sahatiya and coworkers developed a CNT/pencil-eraser-based pressure sensor, the sensitivity of which was 0.135 MPa^−1^ [16]. Karimov et al. reported rubber/MWCNT flexible resistive tensile load sensors and observed a 1.37 times average increase in resistance with an increase in force up to 0.0045 N [17]. N. Jiang and co-workers fabricated, characterized, and tested the piezoresistive and conductive properties of silicone/CNT sandwich-type composites. They reported that the tensile stiffness drops from 196.35 ± 15.3 kPa to 49.58 ± 6.00 kPa due to intrinsic structural change for second loading of the composite [18]. Rekha et al. presented a diaphragm-based square-bossed CNT piezoresistive pressure sensor for biomedical applications. They achieved a sensitivity of 27.82 mV/kPa in the pressure range from 0 to 5 kPa with a non-linearity error of 0.27% [19]. Zhu and co-workers demonstrated graphene-based piezoresistive pressure sensors and by performing electromechanical measurements, they achieved a gauge factor of 1.6 in the dynamic range of 0 to 700 mbar [20]. In 2020, B. Lv et al., prepared a highly sensitive (0.79 kPa^−1^) pressure sensor by coating polyurethane conductive sponge with graphene oxide (GO). The sensitivity of the sensor was 0.79 kPa^−1^ [21]. A CNT—Cu_2_O composite pressure sensor was presented by Karimov et al. [22]. The average thickness and diameter of the pellet were 4 and 10 mm, respectively. A 3.3 times decrease in the resistance was observed as the pressure increased from 0 to 37 kN/m^−2^.

Literature study shows that researchers and scientists focused on reinforcement, electrical, and optical properties of the CNT–graphene composites. However, limited research work has been extended to investigate the role of thickness and fabrication pressure on the CNTs, graphene, and its composite-based piezoresistive pressure sensors. The combination of graphene and CNTs can share the advantages of their unique features that may have a better impact on the piezoresistive properties of the pressure sensors and other electronic devices. In [23], we compared the sensitivities and percent decrease in impedances and resistances of composite samples with pure graphene and CNT-based samples. In continuation with our previous study, in this work, we investigated the effect of thickness and fabrication pressure on the response of the CNT–graphene composite-based pressure sensors with increase and decrease of the external uniaxial applied pressure. We have used a cost-effective and novel approach to investigate the effect of thickness and fabrication pressure on the response of the CNT–graphene composite-based pressure sensors. We believe that this approach will boost up the real-life applications of CNTs, graphene and its composite-based pressure, and other electronics devices in the fields of nano- and sensing technology.

## 2. Materials, Sample Fabrication, and Experimental Setup

### 2.1. Materials

MWCNTs and graphene nanopowders were commercially purchased from Sun Nanotech Co. Ltd., Jiangxi, China. According to the supplier, the purity, range of lengths, and outer diameters of the multi-walled carbon nanotubes (MWCNTs) are >90%, 1–10 µm, and 10–35 nm, respectively. The thickness range and area size of the graphene are 5–20 nm and 10 × 10 µm, respectively. The materials were used for the sample’s fabrication as received without further purification.

### 2.2. Samples Fabrication

**Figure 1:**
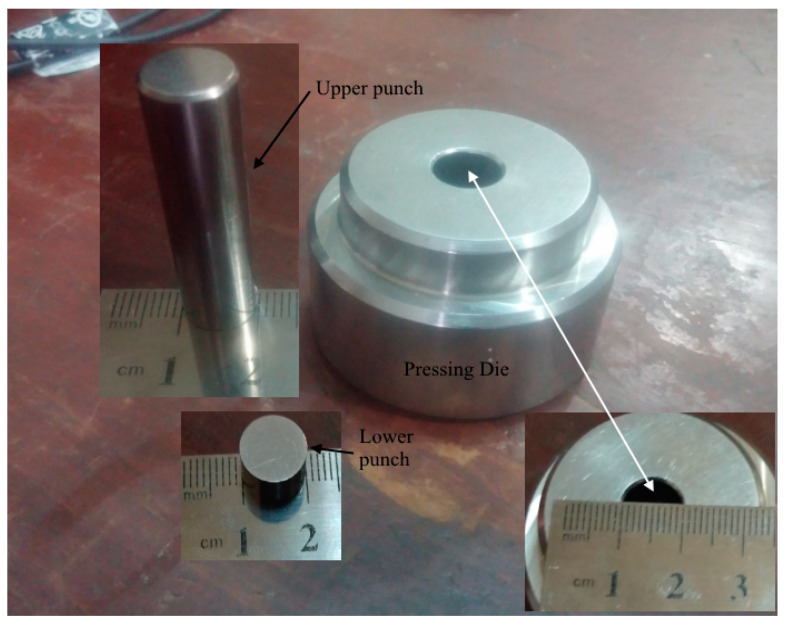
Stainless steel pressing die with inner diameter of 10 mm along with the lower punch for closing the bottom of the die.

Electronic analytical balance (Model: KERN ALS 220-4) was used to accurately measure the amount of graphene and CNT nanopowder. A fine composite of graphene and CNT nanopowder was prepared by blending the material in a mortar and pestle very carefully. The analytical balance has automatic internal adjustment with shockproof construction and accurately works under laboratory conditions in normal temperature. The blend of nanopowder was then poured into a thick-walled cylinder with inner diameter of 10 mm. The bottom of the cylinder was closed with a well-fitted stainless-steel punch, as depicted in Figure 1.

**Figure 2:**
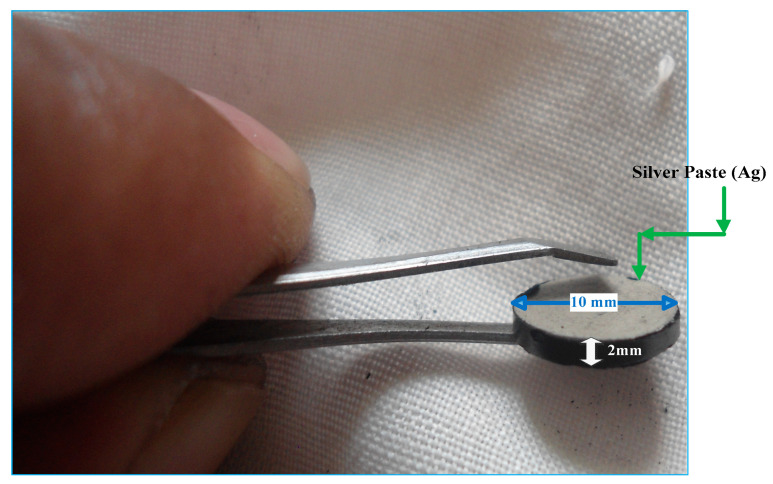
Fabricated sensor of 2 mm thickness and 10 mm diameter with silver paste (Ag) on both sides.

The composite materials were then pressed by hydraulic press to make the sample more durable (Figure 2). The samples were then ejected carefully from the pressing die and then both sides of the samples were covered with silver paste (Ag) and attached to low-resistance electrical contacts. The sandwich-type pressure sensitive sensor (Ag/sample/Ag) is given in Figure 2.

A total of six samples were fabricated for comparison purposes. Three samples of the same thickness of 2 mm were fabricated at different pressures, while the remaining three samples of different thicknesses were fabricated at the same pressure. The exact values of the thicknesses and fabrication pressures of all the samples, along with detailed discussion are given in Section 3.2 and Section 3.3.

### 2.3. Experimental Setup and Measurements

**Figure 3:**
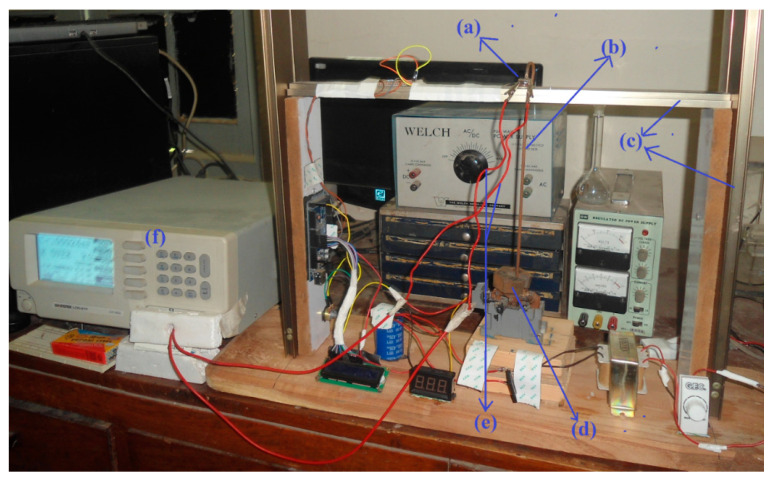
Experimental setup: (**a**) fabricated sensor, (**b**) weight holder, (**c**) metallic support, (**d**) weight, (**e**) test lips of the LCR meter connected with the terminals of the fabricated sensor, (**f**) GW Instek 817 LCR Meter.

To observe the response and investigate the piezoresistive properties of the composite material, the samples prepared in Section 2.2 were installed in the experimental setup shown in Figure 3. Aluminum foil (Al) was used to cover the surface of the pressure sensitive sensor. The purposes of the Al foil are to avoid surface scratching and to use as the terminals of the sample.

**Figure 4:**
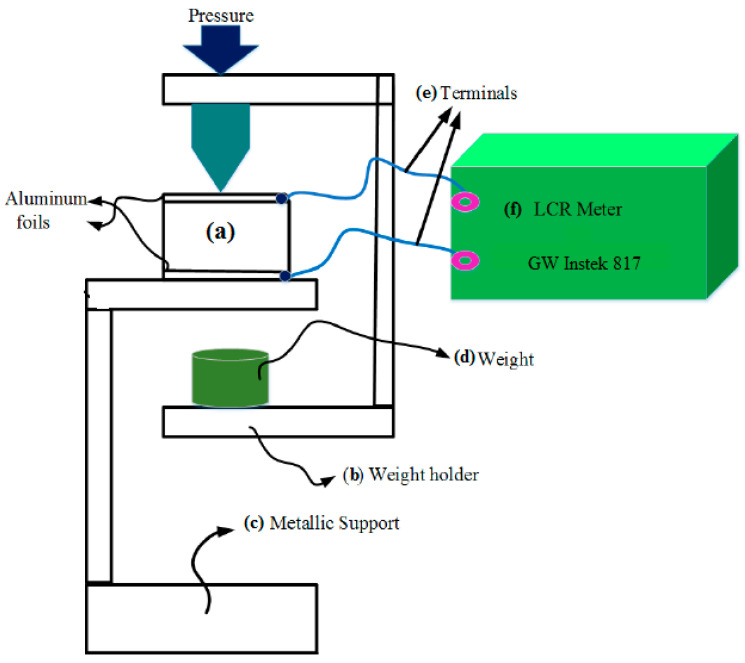
Corresponding schematics diagram of Figure 3.

The corresponding schematic conceptual view of Figure 3 is given in Figure 4. The experimental setup consists of metal support, weight, weight holder, GW Instek 817 LCR meter, and the prepared pressure-sensitive sensor. Each sensor was placed on metal support (Figure 4a). Silver paste (Ag) is considered as part of the sample. Therefore, it is not shown in the figure separately.

One end of the weight holder was placed on the sensor, while the weight, held by the lower end of the weight holder, was changed to vary the pressure accordingly. The terminals of the fabricated sensors were connected with test clips of the GW Instek 817 LCR meter. The measurement range, test signal levels, test speed, dissipation factor, test frequency, and basic accuracy of the LCR meter are 0.00001 Ω–99,999 kΩ, 5 mV–1.275 Vrms, 68 ms, 0.0001–9999, 12 Hz–10 kHz, and 0.05%, respectively. At 0 kg, the initial resistance of the fabricated samples was noted and then the pressure was varied by changing the weight by the lower end of the weight holder. The weight was increased as 0.1, 0.15, 0.2, 0.3, 0.4, 0.5, and 0.6 kg. The weights were then converted to the corresponding pressure by using the standard expression P = W/A, where A is the cross-sectional area of the fabricated sensor, P is the pressure, and W is the weight held by the weight holder. The change in resistance R of the fabricated samples with the change in external uniaxial applied pressure was noted from the display readings of the high-precision GW Instek 817 LCR meter in ambient air at normal temperature.

## 3. Results and Discussion

### 3.1. Scanning Electron Microscopy (SEM)

**Figure 5:**
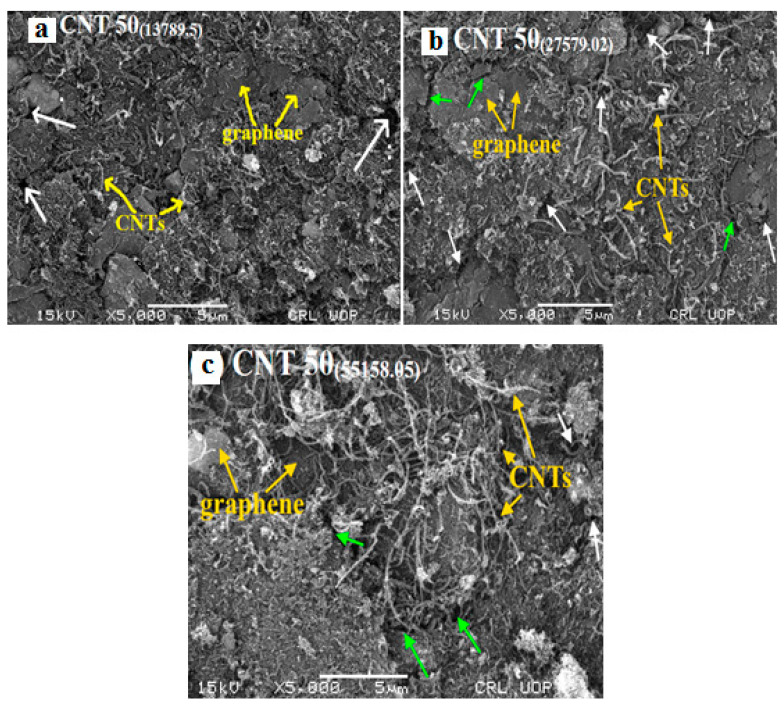
SEM images of the fabricated sensors with different fabrication pressure of (**a**) 13,789.5, (**b**) 27,579.02, and (**c**) 55,158.05 kNm^−2^.

The surface morphology of the fabricated sensors was examined by scanning electron microscopy. Scanning electronic microscopic images of the fabricated sensors with different fabrication pressures of 13,789.5, 27,579.02, and 55,158.05 kNm^−2^, are shown in Figure 5. The SEM images of all the fabricated sensors have the same magnification scale bar of 5 µm. The SEM images of the fabricated sensors with different thicknesses of 1 mm, 2 mm, and 4 mm (CNT501 mm, CNT502 mm, CNT504 mm) are not provided here as changes in thicknesses had no significant effect on surface morphology of the samples, i.e., the surface morphology of the sensors fabricated with different thicknesses were almost similar to Figure 4b. From Figure 5a–c, it can be observed that graphene and CNTs in the composite fabricated sensors are not distributed uniformly. Some voids and pores (white arrow in Figure 5), and cracks (green arrow in Figure 5) can be observed on the surfaces of sensors. Some of the CNTs are straight, some of them are curved, and some of them are even circular in shape. This strong bending behavior shows the extreme flexibility of CNTs, which makes these materials more suitable for pressure sensors and other electronic devices. It can be observed from the surface morphology of fabricated sensors under the external applied pressure that graphene nanosheets are densified and aligned parallel to each other. The microscopic observation of Figure 5a–c shows that the interfacial distance between CNTs and graphene nanoparticles in Figure 5b appears shorter than Figure 5c but higher than Figure 5a. This may be due to the densification effect in the samples fabricated at a higher pressure. The nanoparticles in the sensor prepared at greater pressure (Figure 5c) seem to be in close contact with each other, which, in turn, lead to a higher conductivity and hence smaller resistance of the samples. Detailed discussion of the effect of fabrication pressure on the resistance of the sensors can be found in Section 3.2.

### 3.2. Resistance Versus Fabrication Pressure

**Figure 6:**
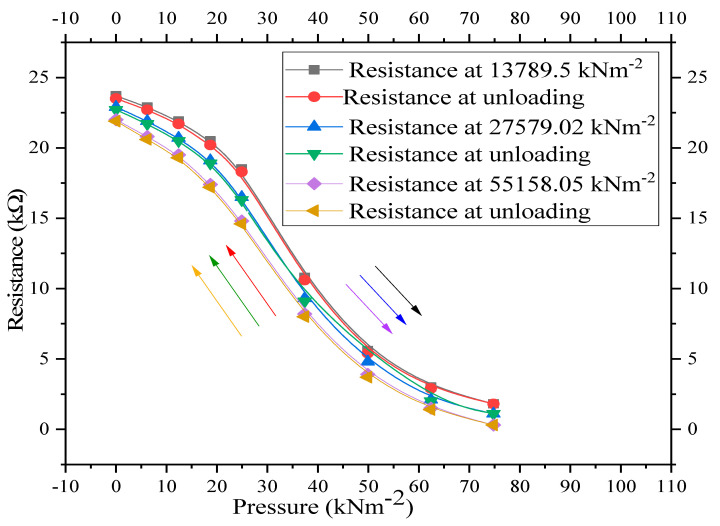
Resistance–pressure characteristics for CNT–graphene composite-based piezoresistive pressure sensors under cyclic loading.

The resistance dependence on the fabrication pressure of the CNT–graphene composite-based piezoresistive pressure sensors under cyclic loading is shown in Figure 6. Three composites of the same thicknesses (2 mm) with 50 wt.% of each ingredient were fabricated at pressures of 13,789.5, 27,579.02, and 55,158.05 kNm^−2^, respectively. The changes in the resistances of the three samples were examined under the same external applied pressure (0–74.8 kNm^−2^). All the samples exhibited a decrease in DC resistance as the external uniaxial applied pressure increased from 0 to 74.8 kNm^−2^. The decrease in resistance with increase in pressure is indicated by downward arrows in Figure 6. It can be observed from Figure 6 that the DC resistance of the composites is inversely proportional to the fabrication pressure. Higher fabrication pressure leads to a lower resistance value and vice versa.

Higher fabrication pressure eliminates pores, which decreases the interfacial distance between nanoparticles, which, in turn, creates more conductive paths in the composites. This leads to higher electrical conductivity and hence smaller resistance. Therefore, the DC resistance of the composites with respect to the fabrication pressure is smaller in the following order: composite fabricated at 55,158.05 kNm^−2^ < composite fabricated at 27,579.02 kNm^−2^ < composite fabricated at 13,789.5 kNm^−2^. However, in the pressure regime, ranging from 24.9 to 49.9 kNm^−2^, a more abrupt decrease in the resistance is observed for the sample fabricated at smaller pressure (13,789.02 kNm^−2^) than the other two samples. This may be attributed to a comparatively larger number of pores in the sample fabricated at lower pressure, i.e., under the same external applied pressure, comparatively large number of pores cause a stronger densification effect in the sample. When the pressure was decreased from 74.8 back to 0 kNm^−2^, the resistance–pressure characteristics did not overlap due to a small hysteresis effect in the CNT–graphene composite-based piezoresistive pressure sensors. Increase in resistance with decrease in pressure back to 0 kNm^−2^ is indicated by upward arrows in Figure 6.

#### Experimental Versus Simulation

The transfer function of the fabricated sensor can be computed either by exponential function, linear function, or polynomial regression approximation [24]. The most appropriate approximation is the polynomial regression model if exponential and linear approximations are not well-fitting to the experimental data as given in Equation (1).

F (X) = A + K_1_X^1^ + K_2_X^2^ + K_3_X^3^ (1)

where F(X) is the predicted outcome value for the polynomial model with regression coefficients from K_1_ to K_3_ and F(X) intercept A. Equation (1) is a polynomial regression model in one variable X. Successively increasing order strategy or forward selection procedure is used to choose the third-order polynomial approximation. A higher-order regression model can be used if higher precision and accuracy is required. However, in our case, the third-order polynomial regression model (Equation (1)) is used to best fit the experimental data of Figure 6.

In our case, the polynomial regression model of Equation (1) transferred to Equation (2) for the pressure–resistance characteristics.

**Figure 7:**
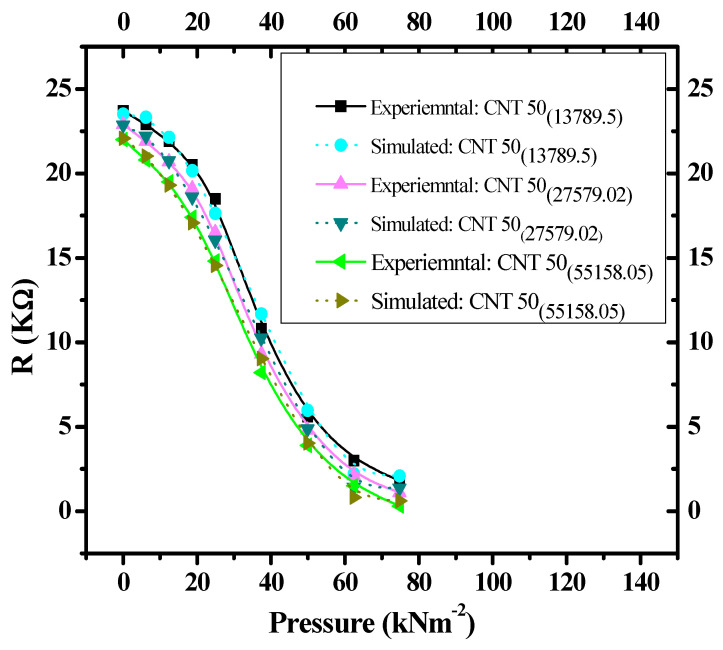
Resistance–pressure characteristics of experimental data (Figure 6) and simulated results (Equation (2)) for CNT–graphene composite-based piezoresistive pressure sensors with fabrication pressures of 13,789.5, 27,579.02, and 55,158.05 kNm^−2^.

**Figure 8:**
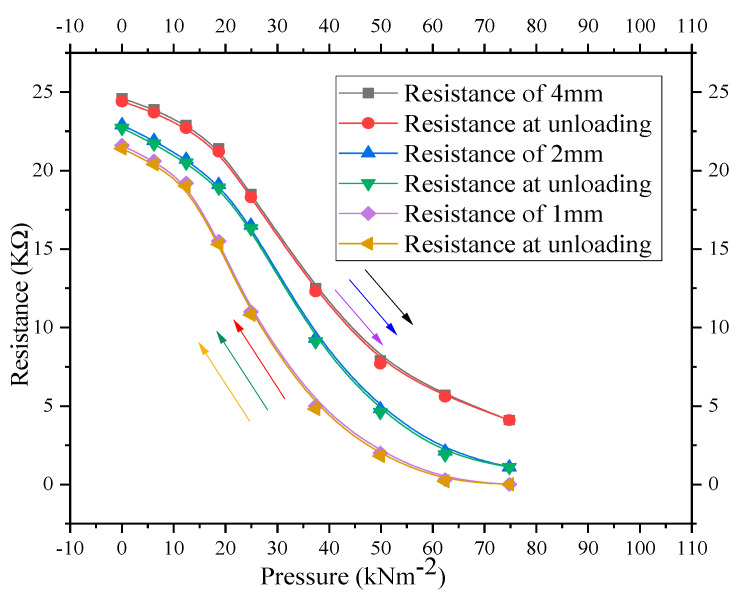
Resistance–pressure characteristics for CNT–graphene composite-based piezoresistive pressure sensors with thicknesses of 1 mm, 2 mm, and 4 mm under cyclic loading.

R = C_o_ + C_1_P^1^ + C_2_P^2^ + C_3_P^3^ (2)

where R is the resistance under the uniaxial external applied pressure P ranging from 0 to 74.8 kNm^−2^. Decrease in resistance R was observed with increase in external uniaxial pressure P as indicated by the downward arrows in Figure 7 and Figure 8. C_o_ is the intercept, which represents the initial resistance R_o_ of the piezoresistive pressure sensors with no pressure. In our case, the general variables X^1^, X^2^, and X^3^ of Equation (1) are transferred to corresponding specified pressure variable P^1^, P^2^, and P^3^, of Equation (2), respectively. C_1_, C_2_, and C_3_ are the pressure factors or fitting parameters and P is the uniaxial external pressure applied on the fabricated sensors. The computed value of C_o_, C_1_, C_2_, C_3_ for the three sensors are given in Table 1.

The experimental data (Figure 6) and simulated results (Equation (2)) for the three composites are shown in Figure 6. For the piezoresistive pressure sensors fabricated at pressures of 13,789.5, 27579.02 and 55,158.05 kNm^−2^, the experimental data (Figure 6) deviates from simulated results (Equation (2)) by 1.4, 2.4 and 2.7%, respectively. Experimental data (Figure 6) and simulated results (Equation (2)) were in excellent agreement with each other, as shown in Figure 7. For the fabricated pressure sensors at pressures of 13,789.5, 27,579.02, and 55,158.05 kNm^−2^, simulated results deviate from the experimental pressure–resistance values by only 1.4, 2.4 and 2.7%, respectively. The amount of deviation of all the experimental characteristics from the simulated curves was calculated by using Equation (3) [25].

(3) $$\%\;\mathrm{Deviation}=\frac{\mathrm{Theoretical}\;\mathrm{value}\;-\;\mathrm{Experimental}\;\mathrm{value}}{\mathrm{Theoretical}\;\mathrm{value}}*100$$

The simulation results are based on curve-fitting techniques or regression analysis. They examine the relationship between one or more predictors and response variables. In our case, the predictor is the external uniaxial pressure P applied on the fabricated samples while the response variable is the resistance R that is observed to be decreased with the increase in predictor. The experimental data represent a material property. If the data is noisy and the material property is dependent upon the applied variable (pressure-dependent resistivity in our case), then we prefer not to use this data directly in further analyses. Such noisy input data can often cause solver convergence difficulties. If we instead approximate the data with a smooth curve, then the convergence of the model can be improved, and we will also have a simple function to represent the material property.

### 3.3. Resistance Versus Thickness of the Composite

The change in DC resistance with thickness variations of the composite under cyclic loading is shown in Figure 8. For the results to be compared, three composites of different thicknesses (1 mm, 2 mm, and 4 mm) with the same CNT and graphene contents (50 wt.% of each ingredient) were fabricated at the same pressure (27,579.02 kNm^−2^). A decrease in DC resistance is observed with increase in external applied pressure from 0 to 74.8 kNm^−2^ as indicated by downward arrows in Figure 8. The measured value of the DC resistance of each sample is a nonlinear function of the thickness. Under the same external uniaxial applied pressure, resistance hardly decreases as the thickness of the sample increases. This is due to the difficulty in reorientation of the nanoparticles in the thicker composite samples than the thinner samples. The nanoparticle orientation at the piston’s contacts and nearer locations is greater than the orientation at some distance from the pistons. As the thickness of the composite increases, this effect becomes weaker as large amounts of particles are far away from the surface and walls of the pistons. Due to the frictional forces between the nanoparticles and on the walls of the cylinder, the top and bottom of the samples are more compressed than the central region. Thus, the external uniaxial applied pressure cannot be equally transferred to every section throughout the composites. Such a pressure inhomogeneity in the compressed powder results in “arching effects”, which causes a decrease in mean coordination number, which, in turn, reduces the contacts between nanoparticles and hence increases the resistance of the thicker sample. Furthermore, larger pressure needs to deform, compress, and densify the thicker composites. Therefore, in thicker samples, even under a high external applied pressure, a small increase in charge carrier concentration may not completely fill the localized energy states present between the higher occupied molecular orbital (HOMO) and lower un-occupied molecular orbital (LUMO). Higher localized energy states between HOMO–LUMO levels may lead to smaller electrical conductivity and hence higher resistance of the composites, as shown in Figure 8. An increase in resistance with a decrease in pressure back to 0 kNm^−2^ is indicated by upward arrows in Figure 8. When the pressure was decreased from 74.8 back to 0 kNm^−2^, the resistance–pressure characteristics did not overlap due to a small hysteresis effect in the composite material.

The resistance of the sensor elements can be calculated by Equation (4) [26].

(4) $$R=\frac{d}{\sigma A}$$

where σ is the conductivity, d is the thickness, and A is the cross-sectional area of the sensor element. According to the percolation theory, the average conductivity of a single component (piezoresistive pressure sensor in our case) can be calculated by Equation (5) [26].

(5) $$\sigma =\frac{1}{LZ}$$

where Z is the resistance of the path with lower average resistance and L is the concentration of the particle in multicrystalline disordered carbonaceous material. As the external uniaxial pressure increases, the density of the sensor element increases, which increases the nanoparticle concentration (L). Increase in L causes a reduction in Z. Therefore, the electrical conductivity increases and hence a decrease in the resistance of the composites is observed in resistance–pressure relationships, as shown in Figure 6 and Figure 8.

#### Experimental Versus Simulation

**Figure 9:**
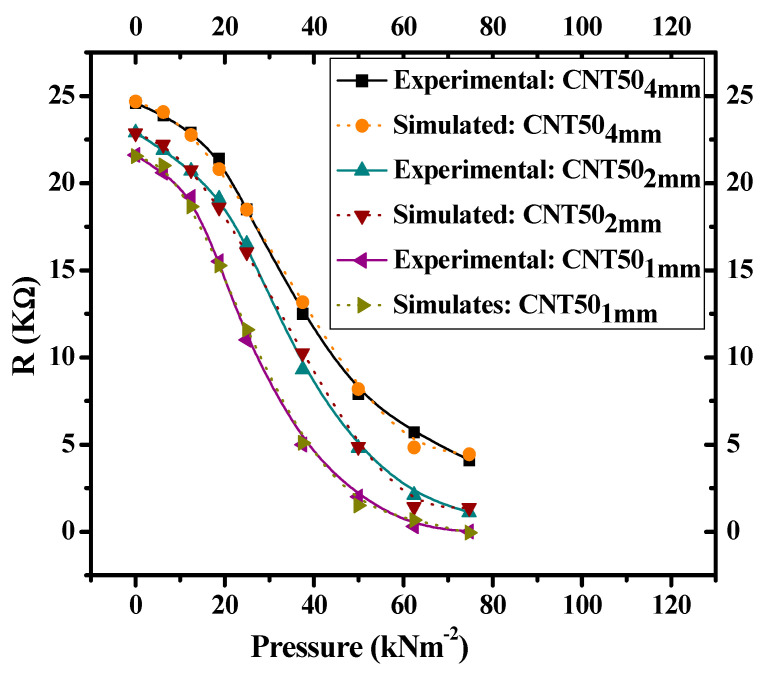
Resistance–pressure characteristics of experimental data (Figure 8) and simulated results (Equation (2)) for CNT–graphene composite-based piezoresistive pressure sensors with thicknesses of 1, 2 and 4 mm.

Since, the shapes of the resistance–pressure relationships of Figure 8 are almost identical to the characteristics shown in Figure 6; therefore, the third-order polynomial functional approximation (Equation (2)) can be applied to the characteristics shown in Figure 8. The experimental data (Figure 8) versus the corresponding simulated results (Equation (2)) for the composites with different thicknesses of 1 mm, 2 mm, and 4 mm are shown in Figure 9.

Under the same external uniaxial applied pressure, experimental data (Figure 8) deviate from simulated results (Equation (2)) by 4.2, 2.4, and 0.45% for composites fabricated with thicknesses of 1, 2 and 4 mm, respectively. The amount of deviation of all the experimental characteristics from the simulated curves are in acceptable range, which was calculated by using Equation (3) [24].

## 4. Conclusions

MWCNT–graphene composite-based piezoresistive pressure sensors were fabricated using the mortar and pestle/hydraulic press technique in the form of pellets. For comparison purposes, three samples with equal CNT and graphene contents and different fabrication pressures of 13,789.5, 27,579.02, and 55,158.05 kNm^−2^; and three more samples with 50 wt.% of each ingredient and different thicknesses of 1, 2 and 4 mm were produced, respectively. It was observed that all the samples prepared with different fabrication pressures show a decrease in resistance with an increase in external applied pressure. However, higher fabrication pressure leads to a lower resistance value of the fabricated sensors, i.e., the DC resistance of the composites is inversely proportional to the fabrication pressure. Besides this, it was observed that thicker samples have smaller electrical conductivity and higher resistance than the thinner samples under the same external uniaxial applied pressure. The effect of the pressure and densification effect on the pores, voids, and particle contacts in the materials were discussed. Percolation theory was used to describe the conduction mechanism in the piezoresistive pressure sensors. The resistance–pressure characteristics were simulated and compared with experimental data. Experimental data deviated from simulated results by 1.4, 2.4 and 2.7% for the composites fabricated at different pressures of 13,789.5, 27,579.02 and 55,158.05 kNm^−2^, respectively. Under the same external uniaxial applied pressure, experimental data deviated from simulated results by 4.2, 2.4 and 0.45% for the composites fabricated with thicknesses of 1, 2, and 4, respectively. For all the samples, experimental data shows excellent agreement with the simulated results.

## Figures and Tables

**Table 1:** Values of the intercept A and pressure factors C1, C2, and C3.

Fabricated Sensor	C_o_ (kN^−2^m^2^)	C_1_ (kN^−2^m^2^)	C_2_ (kN^−2^m^2^)	C_3_ (kN^−2^m^2^)
CNT_50 (13,789_._5)_	23.51275	0.0595	−0.01548	1.45137 × 10^−4^
CNT_50 (27,579_._02)_	22.85745	−0.02818	−0.01305	1.28144 × 10^−4^
CNT_50 (55,158_._05)_	22.07015	−0.111	−0.01037	1.07175 × 10^−4^

## Data Availability

Not applicable.
